# Supplementary material for: Integrated analysis of the aqueous humor microbiome and lens capsule transcriptome in high myopia cataract: a pilot study
Source: Front Med (Lausanne). 2026 Jun 16;13:1845205. doi: 10.3389/fmed.2026.1845205 (PMC13314463; doi:10.3389/fmed.2026.1845205)
Supplement: Supplementary file 3 [file Data_Sheet_1.zip › 3.Beta_Diversity/PCoA/C372089/PCoA_binary_jaccard_3D.pdf]

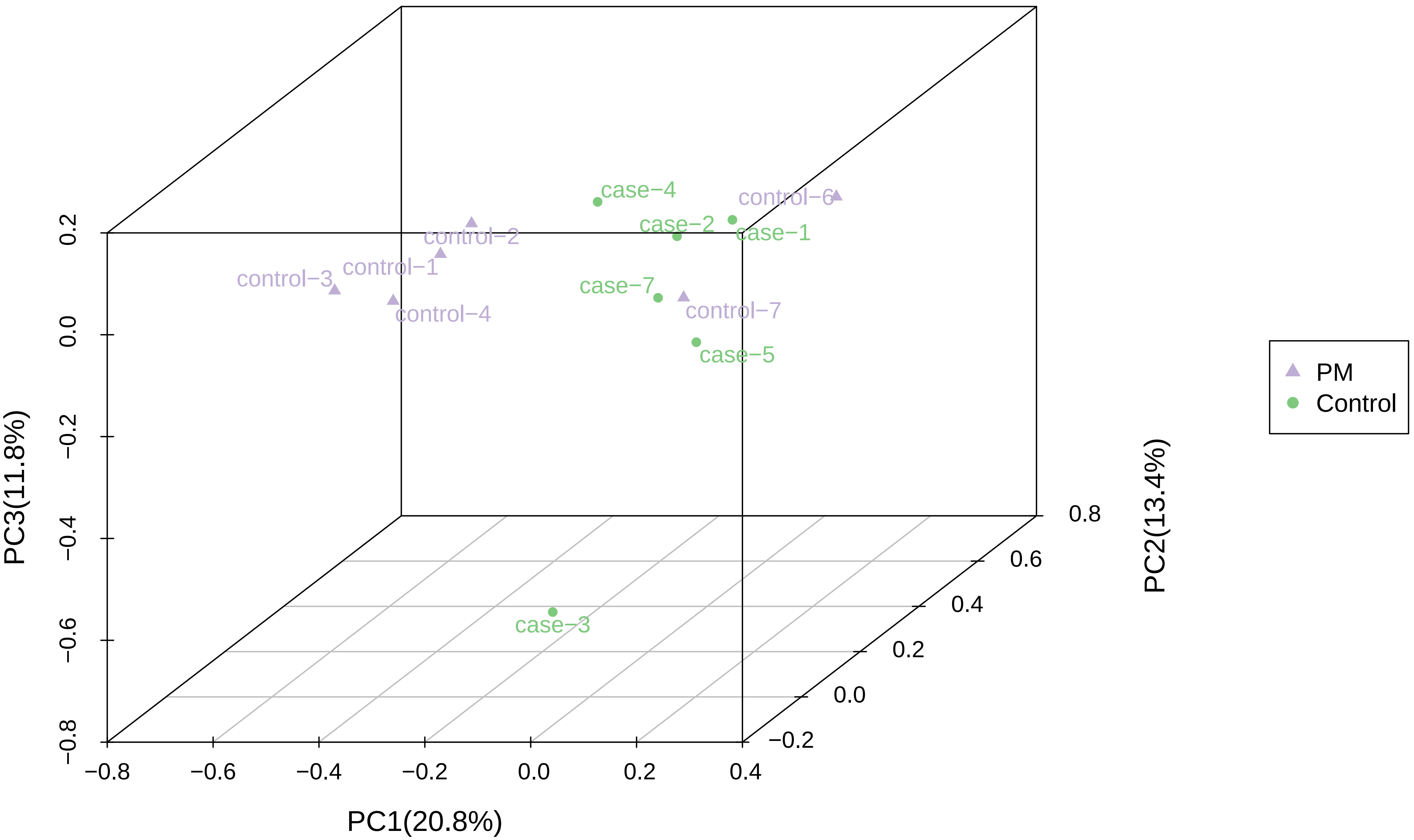

case−4
control−6
case−2
case−1
control−2
control−1
control−3
control−4
case−7
control−7
case−5
case−3
PM
Control
PC1(20.8%)
PC2(13.4%)
PC3(11.8%)
−0.8
−0.6
−0.4
−0.2
0.0
0.2
0.4
0.6
0.8
